# Small Non-coding RNA Expression and Vertebrate Anoxia Tolerance

**DOI:** 10.3389/fgene.2018.00230

**Published:** 2018-07-10

**Authors:** Claire L. Riggs, Amanda Summers, Daniel E. Warren, Göran E. Nilsson, Sjannie Lefevre, W. W. Dowd, Sarah Milton, Jason E. Podrabsky

**Affiliations:** ^1^Department of Biology, Portland State University, Portland, OR, United States; ^2^Department of Psychological and Brain Sciences, Villanova University, Villanova, PA, United States; ^3^Department of Biology, Saint Louis University, St. Louis, MO, United States; ^4^Department of Biosciences, University of Oslo, Oslo, Norway; ^5^School of Biological Sciences, Washington State University, Pullman, WA, United States; ^6^Department of Biological Sciences, Florida Atlantic University, Boca Raton, FL, United States

**Keywords:** miRNA, small ncRNA, anoxia, vertebrate extreme tolerance, epaulette shark, crucian carp, leopard frog, western painted turtle

## Abstract

**Background:** Extreme anoxia tolerance requires a metabolic depression whose modulation could involve small non-coding RNAs (small ncRNAs), which are specific, rapid, and reversible regulators of gene expression. A previous study of small ncRNA expression in embryos of the annual killifish *Austrofundulus limnaeus*, the most anoxia-tolerant vertebrate known, revealed a specific expression pattern of small ncRNAs that could play important roles in anoxia tolerance. Here, we conduct a comparative study on the presence and expression of small ncRNAs in the most anoxia-tolerant representatives of several major vertebrate lineages, to investigate the evolution of and mechanisms supporting extreme anoxia tolerance. The epaulette shark (*Hemiscyllium ocellatum*), crucian carp (*Carassius carassius*), western painted turtle (*Chrysemys picta bellii*), and leopard frog (*Rana pipiens*) were exposed to anoxia and recovery, and small ncRNAs were sequenced from the brain (one of the most anoxia-sensitive tissues) prior to, during, and following exposure to anoxia.

**Results:** Small ncRNA profiles were broadly conserved among species under normoxic conditions, and these expression patterns were largely conserved during exposure to anoxia. In contrast, differentially expressed genes are mostly unique to each species, suggesting that each species may have evolved distinct small ncRNA expression patterns in response to anoxia. Mitochondria-derived small ncRNAs (mitosRNAs) which have a robust response to anoxia in *A. limnaeus* embryos, were identified in the other anoxia tolerant vertebrates here but did not display a similarly robust response to anoxia.

**Conclusion:** These findings support an overall stabilization of the small ncRNA transcriptome during exposure to anoxic insults, but also suggest that multiple small ncRNA expression pathways may support anoxia tolerance, as no conserved small ncRNA response was identified among the anoxia-tolerant vertebrates studied. This may reflect divergent strategies to achieve the same endpoint: anoxia tolerance. However, it may also indicate that there are multiple cellular pathways that can trigger the same cellular and physiological survival processes, including hypometabolism.

## Introduction

Only a handful of vertebrates from diverse lineages have evolved tolerance of extended periods of time without oxygen (anoxia) (Bickler and Buck, 2007). Comparing how these few anoxia-tolerant vertebrate species survive in habitats that are regularly depleted of oxygen provides insight into the evolution of anoxia tolerance, and may uncover unique cellular and genetic mechanisms supporting this rare ability. A canonical component of surviving anoxia is profound metabolic depression; quickly lowering the ATP demand of the organism to meet the decreased supply of ATP under anoxia is critical to surviving long periods without oxygen. Identifying the mechanisms used by animals to coordinate physiological changes associated with entry into and exit out of hypometabolism is important to understanding extreme anoxia tolerance. A recent surge in small non-coding RNA (small ncRNA) research has revealed that small ncRNAs are intricately involved in cell and organismal biology (Morris and Mattick, 2014). Known gene-regulatory functions in a myriad of physiological responses including metabolism, development, stress-response, and normal brain function, make small ncRNAs potential coordinators of metabolic depression and survival of anoxia. The high level of conservation for some classes of small ncRNA sequences and functions across taxa (Bartel, 2004) also makes them compelling targets for potentially identifying conserved mechanisms of anoxia tolerance among vertebrates. Here we conduct the first study to compare the identity and expression patterns of small ncRNAs in some of the most anoxia-tolerant vertebrates known.

Both the independent evolution of anoxia tolerance and the range of tolerance levels exhibited throughout Vertebrata provide a unique opportunity for comparative study. Among vertebrates, a few members of the poikilothermic classes (fish, amphibians, and reptiles) have evolved extreme anoxia tolerance, while other poikilothermic and homeothermic vertebrates are generally highly anoxia-sensitive. We chose the most anoxia-tolerant representatives of the cartilaginous fishes, bony fishes, amphibians and reptiles for this study (**Table 1**). The epaulette shark (*Hemiscyllium ocellatum*), which inhabits the Great Barrier Reef of Australia, is possibly the most hypoxia-tolerant cartilaginous fish (Nilsson and Renshaw, 2004), yet also displays intermediate levels of anoxia tolerance. Exposed to cyclic changes in oxygen levels, this shark survives repeated bouts of hypoxia and even brief periods of anoxia (about 2 h at 19°C), before losing the righting reflex (Wise et al., 1998; Renshaw et al., 2002; Dowd et al., 2010). Adult crucian carp (*Carassius carassius*), a bony fish, is extremely anoxia-tolerant. This species inhabits small lakes in northern Europe, where it frequently experiences and survives months of anoxia while overwintering under the ice at temperatures close to 0°C (Nilsson and Renshaw, 2004). The western painted turtle (*Chrysemys picta bellii*) is the most anoxia-tolerant tetrapod (Keenan et al., 2015) and routinely overwinters in ice-covered ponds and streams in the northern United States and southern Canada in water or mud with little to no oxygen (Ultsch, 1989). In the lab, western painted turtles survive over 4 months without oxygen at 3°C (Ultsch and Jackson, 1982; Jackson, 2000). The leopard frog (*Rana pipiens*) is one of the most anoxia-tolerant amphibians, but is only moderately anoxia tolerant. Like the western painted turtle, the leopard frog also overwinters under ice, where they withstand anoxia for several days (Bickler and Buck, 2007). At 25°C, leopard frogs survive only 4–5 h without oxygen (Lutz and Reiners, 1997; Knickerbocker and Lutz, 2001). Since the leopard frog and the epaulette shark are less anoxia tolerant than the carp and turtle, they represent intermediates on the spectrum between highly anoxia-tolerant and anoxia-sensitive species (Bickler and Buck, 2007). While the habitats and physiology of each species vary, all of these species employ profound metabolic depression in order to survive anoxia (Lutz et al., 1996; Milton et al., 2003; Nilsson and Renshaw, 2004), and therefore, may employ similar mechanisms at the cellular and genetic levels, such as with small ncRNAs.

**Table 1:** Anoxia tolerance and sampling parameters for each species.

Anoxia tolerance level	Class	Organism	Tissue Sampled	Sampling time points (A = anoxia; R = recovery)	Sampling Temp. °C	Tolerance at sampling temp
Highly anoxia-tolerant	Reptilia	Freshwater turtles *Chrysemys picta bellii*	Telencephalon	(A) Normoxia	3	5 months (Jackson, 2000)
				(B) 1 week A		
				(C) 1 week A + 1 week R		
Highly anoxia-tolerant	Osteichthyes	Crucian carp *Carassius carassius*	Whole brain	(A) Normoxia	7	Months (Holopainen et al., 1997)
				(B) 1 week A		
				(C) 1 week A + 6 days R		
Moderately anoxia-tolerant	Amphibia	Leopard frog *Rana pipiens*	Whole brain	(A) Normoxia	25	4–5 h (Lutz and Reiners, 1997)
				(B) 1 h A		
				(C) 1 h A + 1 h R		
Moderately anoxia-tolerant	Chondrichthyes	Epaulette shark *Hemiscyllium ocellatum*	Cerebellum	(A) Normoxia	16–18	2.36 h (Dowd et al., 2010)
				(B) A + 24 h R + 50 min anoxia		
				(C) A + 2 h R		

*The epaulette shark samples available for this study are tissues saved from a previous study. Epaulette shark were exposed to anoxia until they lost their righting reflexing, at which time they were brought back to normoxic conditions. Sample ‘B’ is from 2 anoxic exposures, separated by 24 h of recovery. There are no samples available from immediately after 1 anoxic episode, but similar and important gene expression patterns are expected during the 2nd anoxic exposure*.

Small ncRNAs are short (<200 nucleotides) single-stranded RNAs that do not typically code for protein products, yet retain the ability to substantially influence gene expression and cell physiology (Gomes et al., 2013). Some classes of small ncRNAs are degradation products of longer RNAs (Röther and Meister, 2011), which may make them particularly suited for rapid employment in the cell. microRNAs (miRNAs), the most thoroughly studied class of small ncRNAs, are ubiquitous in the cell, regulate a large portion of the genome (Friedman et al., 2009), and are highly conserved in sequence and function across taxa (Bartel, 2004). miRNAs can alter gene expression precisely and rapidly by complementarily binding to target mRNAs (Bartel, 2004). miRNAs are globally stable, with half-lives ranging from 28 to 220 h (Ruegger and Grobhans, 2012). Constitutive expression is important to maintain normal cell and organismal function such as metabolism, development and regulation of gene expression (Ambros, 2004; MacFarlane and Murphy, 2010). In the brain a diversity of miRNAs are involved in normal development, as well as function, including neuronal plasticity and synapses (Kosik, 2006; Shao et al., 2010).

Exposure to stress induces changes in miRNA expression in stress-tolerant and stress-sensitive organisms, alike. miRNAs are differentially expressed in mouse and mammalian models in response to hypoxia and ischemia (occlusion of blood flow, leading to tissue hypoxia or anoxia) (Kulshreshtha et al., 2007a,b, 2008), as well as ischemic preconditioning (brief non-lethal exposure to ischemia that extends the organism’s survival under a subsequent exposure) (Lee et al., 2010). Other non-miRNA small ncRNAs are also rapidly being discovered, many of which also regulate gene expression. An extensive study has recently characterized small ncRNA expression patterns in anoxia-tolerant embryos of the annual killifish *Austrofundulus limnaeus* (Riggs and Podrabsky, 2017), henceforth referred to as annual killifish. Known and novel stress-responsive sequences respond to exposure to anoxia and aerobic recovery in anoxia-tolerant annual killifish embryos. In highly anoxia-tolerant and metabolically active embryonic stages, exposure to anoxia induced differential expression of mitosRNAs, a recently described class of small ncRNAs derived from the mitochondrial genome (Riggs and Podrabsky, 2017).

The aim of this study is to characterize the small ncRNAs present under normoxia and in response to anoxia and recovery in brain tissue of the most anoxia-tolerant vertebrates. Since the brain is one of the most anoxia-sensitive vertebrate organs and has a highly conserved function across taxa, we reasoned that studying this tissue could reveal important conserved small ncRNA expression patterns. We hypothesized that all species would share a common small ncRNA response to anoxia that represents shared molecular pathways that must be regulated in a similar manner across all species. We also hypothesized that mitosRNAs identified in the anoxia-tolerant embryos of the annual killifish would be identified in the other anoxia-tolerant vertebrates and exhibit similar changes in expression in response to anoxia and recovery.

## Materials and Methods

### Animal Husbandry, Experimental Design and Sampling

All species were sampled as adults. Animals were sampled prior to, during, and following exposure to anoxia (re-oxygenation). Sampling conditions, temperature and duration of anoxia, were determined for each species by the researcher with expertise in that particular species. Sampling conditions were chosen based on the natural habitat of the animal, feasibility of the experiment, and comparability to other anoxia studies conducted on each species. Preference was given to conditions for which physiological and other biochemical data were already available. Sampling information is provided in **Table 1**. Four biological replicates (*n* = 4) were generated for each treatment group of each species.

#### Epaulette Shark

Epaulette shark brain tissue samples used in this study were collected for a prior study (Dowd et al., 2010). Collection of specimens was conducted under the Great Barrier Reef Marine Parks Authority permits G07/24973.1 and G07/23338.1. Briefly, sharks (*n* = 42, mean mass = 356 ± 21 g) of both sexes were caught by hand at the Heron Island Research Station and transported in mesh bags to holding tanks where they were maintained under natural winter conditions in 5000 L glass tanks, 1 animal per tank. The epaulette sharks were housed in winter conditions in seawater from the ocean with temperature ranging from 16.5–20°C, salinity of 35.5–37 ppt, and the light cycle set to mimic a natural winter photoperiod. Water changes of 20% volume were conducted each day, exchanging the water for fresh seawater. Anoxia experiments were initiated about 3.5 h after collection. The sharks were not fed prior to anoxia. For anoxia experiments animals were held in 65 L glass tanks, 1 animal per aquarium, at ambient temperature in seawater from the ocean. Compressed nitrogen gas was bubbled into the water to create an anoxic environment (0.1% air saturation, defined as 0.03 mg O_2_/L) (Dowd et al., 2010). Brain tissue samples for the following treatments were included in this study: A1 + A2 (anoxia till loss of righting + 24 h normoxic recovery + 50 min anoxia); C1 + 2 h (2 h in hold tank); A1 + 2h (anoxia until loss of righting reflex + 2 h normoxic recovery). At the appropriate sampling times, animals were euthanized with an overdose of benzocaine (∼80 – 100 mg/l). Within 4 min of euthanasia, half of the cerebellum was quickly dissected out and flash frozen in liquid nitrogen. Samples were stored at -80°C until further processing. Samples were shipped overnight on dry ice to Portland State University for RNA extraction.

#### Crucian Carp

The crucian carp sampled for the current experiment (*n* = 27, mean mass = 28.8 ± 10.6 g) of both sexes were obtained from Tjernsrud pond, Oslo, Norway, in November 2014. Collection of animals and the subsequent experiments were approved by the Norwegian Animal Research Authority (approval no. 8400). Crucian carp were collected in autumn, as this is the best time of the year for collection. The animals were kept in a 750 L holding tank in the aquarium facilities at the Department of Biosciences, University of Oslo. The holding tanks were supplied with aerated and de-chlorinated Oslo city tap water at 6–9°C, and subjected to a 12L:12D light cycle. The fish were maintained under stable conditions and were fed daily with commercial carp food, but food was withheld 24 h prior to and during any experiments. Experiments were conducted about 180 days after collection, in May 2015. Two identical cylindrical dark tanks (25 L) were held at 7°C and equipped with flow-through of water and aeration, one tank serving as the normoxic control tank and the other as the anoxia/re-oxygenation tank (Ellefsen et al., 2009). Twenty-five fish were acclimated in each tank for approximately 24 h prior to experiments. Not all 25 fish from the normoxic tank were sampled, but having the same number of fish in each tank made the environment equal for the two groups, except for the oxygen level. Oxygen saturation and temperature were monitored daily using an Oxi3310 oxygen meter (WTW, Weilheim, Germany). The normoxic tank remained above 95% of air saturation for the entire exposure period. In the anoxic tank, oxygen levels were maintained below the detection limit of the oxygen meter (0.01 mg/L or 0.1% saturation) and were considered anoxic (Nilsson, 1989). To obtain the anoxic water, nitrogen gas was bubbled directly into the anoxic tank. After the anoxic period, water flow was restored in both tanks, and nitrogen bubbling was replaced with air bubbling, thus resulting in complete re-oxygenation of the water. The normoxic control group and the anoxia group were sampled after 6 days (N6 and A6, respectively). The remaining groups were sampled after 6 days of anoxia plus 1 day of re-oxygenation (A6R1) and 6 days of re-oxygenation (A6R6). At each sampling point, the fish were given a sharp blow to the head, followed by cervical transection. The fish were kept in a vertical orientation to avoid blood flow to the head. Dissection proceeded by opening the cranium from the top and removing the brain as one piece. The brain tissues were immediately placed in RNAlater^TM^ (Ambion Inc., Austin, TX, United States) and frozen at -20°C for storage until shipping to Portland State University for RNA extraction.

#### Leopard Frog

Leopard frogs (*n* = 12) were obtained from a commercial supplier (Charles D. Sullivan Co. Inc., Nashville, TN, United States). Animal husbandry and experimental procedures were approved by the Florida Atlantic University Institutional Animal Care and Use Committee (IACUC protocol number A15-11). Animals weighed 30–55 g and were not sexed. Frogs were maintained in 65 L plastic pens, 6 frogs/pen, with constant access to fresh water at 25°C, a 12L:12D light cycle, and daily feeding of live crickets until 5 days prior to conducting experiments. Experiments were conducted 1 week after obtaining frogs. The following experiments were conducted at Florida Atlantic University, with previously established methods (Milton et al., 2003). All sampling was conducted at room temperature (25 ± 1°C). Control animals were removed from the holding tank and sampled. For anoxia exposure, frogs were held in a 2.6 L sealed, humidified polyethylene chamber, 4 frogs/chamber, in dechlorinated water bubbled with positive-flow nitrogen as previously described and validated (Milton et al., 2003). Animals were removed from the chamber after 1 h and allowed to recover under aerobic conditions for 1 h. Frogs were sampled after 1 h of anoxia and after 1 h anoxia followed by 1 h aerobic recovery. Replication was as for the other species, except for recovery where only three individuals survived the treatment. At each sampling time, animals were quickly decapitated. The brain was dissected out in less than 1 min and immediately flash frozen in liquid nitrogen. Samples were stored at -80°C until shipping on dry ice to Portland State University for further processing.

#### Western Painted Turtle

Western painted turtles (*n* = 15, mean mass = 228 ± 17 g) of both sexes were purchased from Niles Biologicals (Sacramento, CA, United States) in October 2014. All procedures involving the turtles were in strict accordance with the recommendations in the Guide for the Care and Use of Laboratory animals of the National Institutes of Health. All animal husbandry and experimental protocols were approved by the Saint Louis University IACUC (protocol number 2198). Turtles were held at 20–23°C in 750 L fiberglass tanks with St. Louis, MO, United States municipal tap water and basking access to broad spectrum lamps (ZooMed 5.0) and a heat source (60 W incandescent bulbs) under a weekly updated natural photoperiod from October to December. Municipal water was partially dechlorinated by bubbling with air. Turtles were fed 3x/week with commercially available turtle feed (Tetra ReptoMin) until used in experiments carried out in February 2015. Turtles were maintained in the lab for approximately 100 days before conducting experiments. All 15 of the turtles were transferred to a black aquarium filled half-way with 20°C water, after which it was sealed with a lid that included a small glass window to permit photoperiod maintenance (8L:16D). The aquarium was also outfitted with bubbling stones on its bottom for either aeration during normoxia or nitrogen bubbling during anoxia exposure. After 1 day at 20°C, the water temperature was decreased by 3°C, and then by 2°C/d afterwards for 7 days until the turtles were finally at the experimental temperature of 3°C.

After 8 weeks of acclimation at 3°C, the lid was removed, the control turtles were sampled (see below for details) and water (3°C) added so that only a 5–10 cm air space existed between the water surface and the lip of the aquarium. A plastic grate was fixed 2 cm below the surface of the water to prevent the turtles from accessing this airspace, after which the air pump aerating the water was replaced with a nitrogen gas cylinder and the aquarium resealed with the lid. The nitrogen gas bubbling displaced all measurable dissolved oxygen (detectable to 0.01 mg/L or 0.1% saturation) in the system within 1 h (YSI DO200 oxygen meter, YSI Inc., Yellow Springs, OH, United States). Turtles were maintained under these anoxic conditions for 7 days, after which the lid was removed and additional turtles were sampled (see details below). The nitrogen cylinder was then replaced with an air pump and the water level decreased back to 50%, permitting the remaining turtles to recover by breathing fresh air for the next 7 days before they were sampled.

All tissue collection involved aseptic tools in a 3°C cold room. For control and recovery treatments, turtles were removed from the experimental chamber and their necks were clamped followed by immediate decapitation using a guillotine. For anoxic animals, necks were clamped underwater to prevent inhalation of air prior to sampling. The brain was removed from the skull and transferred to sterile aluminum foil for further isolation of the telencephalon, which was dissected free of all white matter and dura, separated into hemispheres, and flash-frozen using clamps pre-cooled in liquid nitrogen. This entire sampling procedure was completed within 2–5 min/turtle. Samples were stored at -80°C until shipping overnight on dry ice to Portland State University for subsequent RNA extractions. Immediately after the brain dissection, and with the turtle’s neck still clamped, blood was collected for gas and metabolite analysis (Supplementary Methods and Supplementary Table S1).

### Choosing Samples for Sequencing

In order to balance mass and sex ratios across the treatment groups, equal numbers (where possible) of males and females were chosen from among the available samples. Final distribution of sexes and range of masses did not differ significantly by group. See Supplementary Table S2 for sample metadata. One-way ANOVA was used to determine any difference in sex ratio between control, anoxic, and recovered turtle groups. Student–Newman–Keuls was used as the *post hoc* test, and significance was indicated if *p* < 0.05. Statistical analyses were performed using SigmaPlot 11 (Systat Software, Inc., San Jose, CA, United States). Normal distribution of mass in turtle and carp samples did not differ significantly by group (Shapiro–Wilks, turtle: *p* = 0.1349; carp: *p* = 0.525). The Kruskal–Wallis test confirmed that there were no differences in sex ratios among the groups (turtle: *p* = 0.7303; carp: *p* = 1). All sharks used in the study were male. Distribution of body mass and length did not differ by treatment group, (Kruskal–Wallis, *p* = 0.2319 and *p* = 0.3397, respectively). Frogs were not sexed, but none were obviously female; no eggs were found upon dissection. Mass was not collected for each frog, so no analysis of distribution was performed.

### RNA Extraction

RNA was extracted from brain tissue according to the TRIzol^TM^ reagent (Invitrogen Inc., Carlsbad, CA, United States) manufacturer’s protocol. Tissue samples were quickly weighed and transferred to a tube containing TRIzol^TM^ for immediate homogenization. Homogenates were phase separated with chloroform, and RNA was precipitated from the aqueous phase by overnight incubation in a high-salt solution. Total RNA for each sample was resuspended in 1 mM sodium citrate (pH = 6.4). RNA concentration and purity were determined by measuring absorbance at 260 and 280 nm and calculating the *A*_260_/*A*_280_ ratio, using an Infinite Pro M200 plate reader equipped with a NanoQuant plate (Tecan, San Jose, CA, United States). To assess RNA integrity, RNA was run on a 2% agarose gel, stained with ethidium bromide and visually inspected for distinct bands representing 18S and 28S rRNA subunits. The mean *A*_260_/*A*_280_ ratio was 2.17 ± 0.026 (S.D.). All samples used to prepare sequencing libraries were high quality RNA samples with *A*_260_/*A*_280_ ratios ranging from 2.06 to 2.22 and distinct bands for the 18S and 28S rRNA ribosomal subunits present (Supplementary Table S2). Total RNA was stored at -80°C until small RNA cDNA library preparation.

### Small ncRNA Sample Preparation and Sequencing

Small RNA cDNA libraries for each individual library were prepared using the TruSeq^®^ small RNA sequencing kit (Illumina, Inc., San Diego, CA, United States) and 1 μg total RNA as starting material as previously described (Riggs and Podrabsky, 2017). Briefly, small RNAs were adapter-ligated, reverse transcribed and amplified by PCR to generate cDNA libraries, which were purified and size separated on a 6% polyacrylamide gel. Small RNAs, 15–30 nucleotides in length, were excised from the gel, precipitated in ethanol, and resuspended in 10 mM Tris-HCl (pH = 8.5) and stored at -20°C. Small RNA library validation and sequencing was conducted at the Oregon Health and Science University Massively Parallel Sequencing Shared Resource (OHSU MPSSR). Quality was assessed by running samples on an Agilent Technologies 2100 Bioanalyzer using a DNA-1000 chip, and libraries were quantified by real-time qPCR prior to cluster generation. Samples were sequenced on an Illumina HiSeq 2000 using single end sequencing for 36 or 50 cycles (Supplementary Table S2), depending on other samples available to run at the time. Samples were multiplexed 12 per flow cell lane, each with a unique index to allow for computational de-multiplexing after sequencing. Biological replicates and samples from the same species were distributed across flow cell lanes to eliminate any effect of lane-bias. Bcl2fastq2 version 2.1.17.1.14 was used to initially process the data and generate fasta files for analysis.

### Data Repository

Raw sequence files have been deposited in NCBI Sequence Read Archive (SRA) under the following Bioproject IDs: *A. limnaeus-*PRJNA272154, *C. picta bellii-* PRJNA375813, *H. ocellatum-* PRJNA375814, *C. carassius*- PRJNA375815, *R. pipiens-* PRJNA375816.

### Small ncRNA Sequence Processing and Analysis

Small ncRNA sequences were trimmed to remove adapters and low-quality reads (phred score < 30), using Trimmomatic (version 0.33; Bolger et al., 2014). Reads 15–27 nucleotides long were retained for analysis to capture canonical miRNAs as well as other known and novel classes of small ncRNAs. Where available, sequences were annotated against the genome of the species sampled, or closely related species in the case of the crucian carp: common carp *Cyprinus carpio* (GCA_000951615.1), and *Chrysemys picta bellii* (GCA_000241765.2 Chrysemys_picta_bellii-3.0.3) using CLC Genomics Workbench (version 6.5; CLC bio, Arhus, Denmark). Sequences that aligned with 0 mismatches to their species’ genome were retained for further analysis. Sequences longer than 27 nucleotides were removed. Within each species small ncRNA expression values were normalized for library size to account for differences in sampling depth, or coverage, of a library and to produce expression values on a common scale so that comparisons could be made between treatments. Library size factors were determined and applied with the R Bioconductor package DESeq2, according to established methods (Anders and Huber, 2010). In brief, size factors, representing sampling depth of a library, were determined for each library (corresponding to one replicate). Expression values of small ncRNAs were then divided by the size factor of its library, putting libraries of different samples on a common scale. Expression values were not normalized based on gene length (as is common for mRNA studies) since all genes of small ncRNAs are approximately the same length and small ncRNAs are not annotated in genomes of the species studied. Within each species, small ncRNA sequences whose normalized expression values (counts adjusted for library size) across all samples (all treatments and replicates) summed greater than 4 were established as the catalog of small ncRNAs for that species and retained for subsequent analysis. A minimum normalized count of 4 across all samples within a species was chosen since each treatment within a species is comprised of four replicates, ensuring that at the minimum the sequence would have an average normalized count of 1 in each replicate of a treatment to be included in the catalog. This filtering was performed in order to reduce the high abundance of lowly expressed reads from the dataset. After filtering on expression, resulting catalogs were annotated to known small ncRNAs documented in miRBase (v.19; Griffiths-Jones et al., 2006), RFAM (version 12.1; Nawrocki et al., 2015), and the available mitochondrial genomes. Where available, mitochondrial genomes of the target species were used: *C. carassius* (NC_006291.1) and *C. picta bellii* (Jiang et al., 2016) (NC_023890.1). For *R. pipiens* and *H. ocellatum* mitochondrial genomes of the closest related species available were used, *R. catesbeiana* (Lin et al., 2014) (NC_0222696.1) and *Chiloscyllium griseum* (NC_017882.1), respectively. Annotations were conducted in CLC Genomics Workbench (version 6.5; CLC bio, Arhus, Denmark), allowing for up to 2 mismatches, and 2 additional or missing bases up or downstream of the annotating sequence. For complete lists of sequences identified in each species’ catalog, see Supplementary Table S3. Description and analysis of differentially expressed small ncRNA sequences present in the catalog of each species was performed using R (R Core Team, 2016), version 3.2.1 (2015-06-18 – “World-Famous Astronaut”). Differential expression tests of small ncRNAs were performed across experimental treatments within each species/stage using R Bioconductor package DESeq2 (Love et al., 2014). *P-*values were called using the likelihood ratio test (LRT) with independent filtering on to remove remaining low count sequences (Anders and Huber, 2010). *P-*values were adjusted with the Benjamini-Hochberg multiple comparisons adjustment with a FDR of 10%. Differentially expressed sequences in response to anoxia and recovery within each species were identified based on the following criteria: p adj < 0.05; log_2_ fold change > 1 or <-1; base mean > 25 normalized counts across all samples. Within each species, fold-change was calculated relative to normoxic expression values for analysis of differential expression in response to anoxia and recovery from anoxia. See Supplementary Table S4 for a complete list of differentially expressed sequences and their annotation information.

Within each species, highly differentially expressed small ncRNAs of interest were clustered by expression pattern with Cluster 3.0 (Eisen et al., 1998) for heat-map generation. Data were log transformed and organized into clusters based on *k*-means, using the Euclidean distance similarity metric for 100 runs. Clustered expression patterns were viewed as a heat-map using Prism (version 7; GraphPad Software Inc., La Jolla, CA, United States).

The most abundant sequences during normoxia and anoxia were determined for each species by sorting the sequences by mean normalized expression value from highest to lowest. The top 100 sequences were considered the most abundant sequences for each species.

#### Literature Search

To compare miRNAs to known stress/hypometabolism, ischemia/hypoxia, and ischemic-preconditioning (IP)-responsive miRNAs in the literature we referenced a database of miRNAs known to respond to these conditions (Riggs and Podrabsky, 2017). The top 40 most abundant miRNAs identified in brain tissue of humans and rhesus macaques (Shao et al., 2010) were also compared to the list of known stress-responsive miRNAs and used for comparison to the anoxia-tolerant species included in this study.

## Results

### Total RNA Levels Are Stabilized During Anoxia

Total RNA per mg brain tissue did not differ in response to anoxia and recovery in any species (Supplementary Figure S1), indicating an overall stabilization of RNA levels in response to anoxia for all species. Additionally, within each species, a similar distribution of sequence lengths was represented, with a peak in abundance near 22 nucleotides in length, indicating a predominance of miRNAs (Supplementary Figure S2).

### Catalog of Small ncRNAs Present in Each Species

**FIGURE 1:**
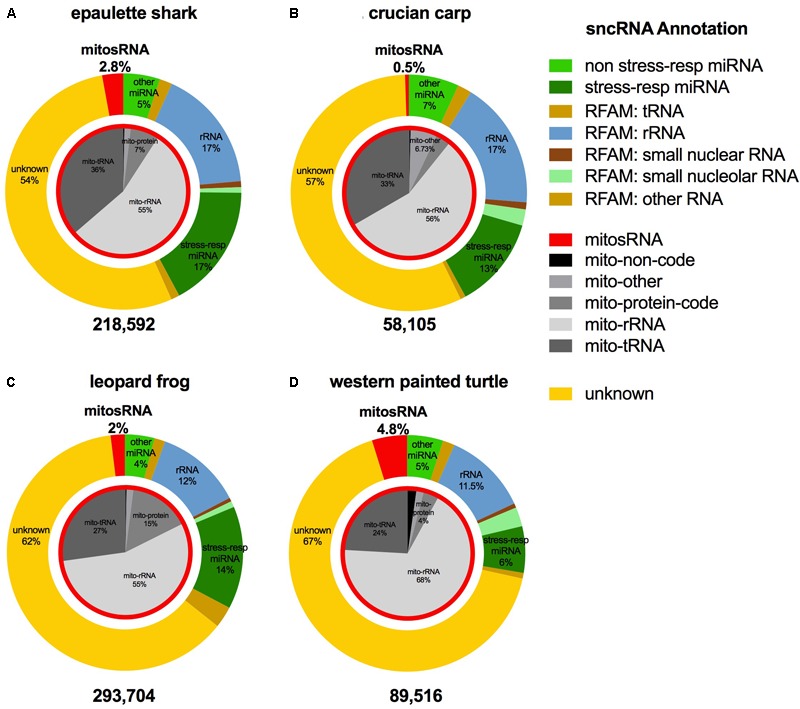
Distribution of the subclasses of small ncRNAs identified in each species. The outer ring (colors) depicts the percent of each annotation category for all small ncRNAs identified in the complete catalog (includes small ncRNAs identified under each treatment) for the species. The inner pie chart (gray scale) represents the annotation location within the mitochondrial genome for mitosRNAs identified in the complete small ncRNA catalog. **(A)** Epaulette shark; **(B)** crucian carp; **(C)** leopard frog; **(D)** western painted turtle.

**FIGURE 2:**
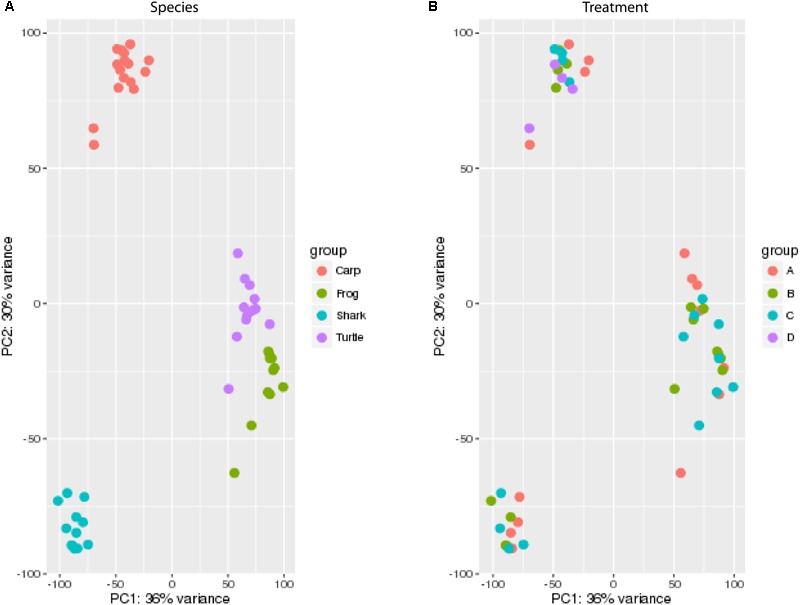
Principal component analysis (PCA) of the catalog of all small ncRNAs for each species. Expression values were normalized by library size across all species for PCA. **(A)** Samples color coded by species (see legend). **(B)** Samples color coded by treatment (see legend). A, normoxia; B, anoxia; C, short recovery; D, long recovery (carp only).

The small ncRNA catalog of each species is comprised of all the small ncRNAs identified under any of the treatments. Diverse small ncRNA subclasses, including sequences annotating to known miRNAs (stress and non-stress-responsive), transfer RNAs (tRNAs), ribosomal RNAs (rRNAs), small nuclear RNAs (snRNAs), small nucleolar RNAs (snoRNAs), mitosRNAs and other types of RNA, were present in each species (**Figure 1**). However, the majority of small ncRNAs identified (69-84%, depending on the species) did not annotate to any previously described RNA and are therefore considered “unknown.” These sequences could be any type of the aforementioned small ncRNAs, or may represent different subclasses yet to be described and characterized. Each species has a unique expression pattern of small ncRNAs as illustrated by species-specific, rather than treatment-specific clustering in a principle component analysis (**Figure 2**).

### Anoxia-Tolerant Vertebrates Similarly Express and Stabilize Stress-Responsive Small ncRNAs

**FIGURE 3:**
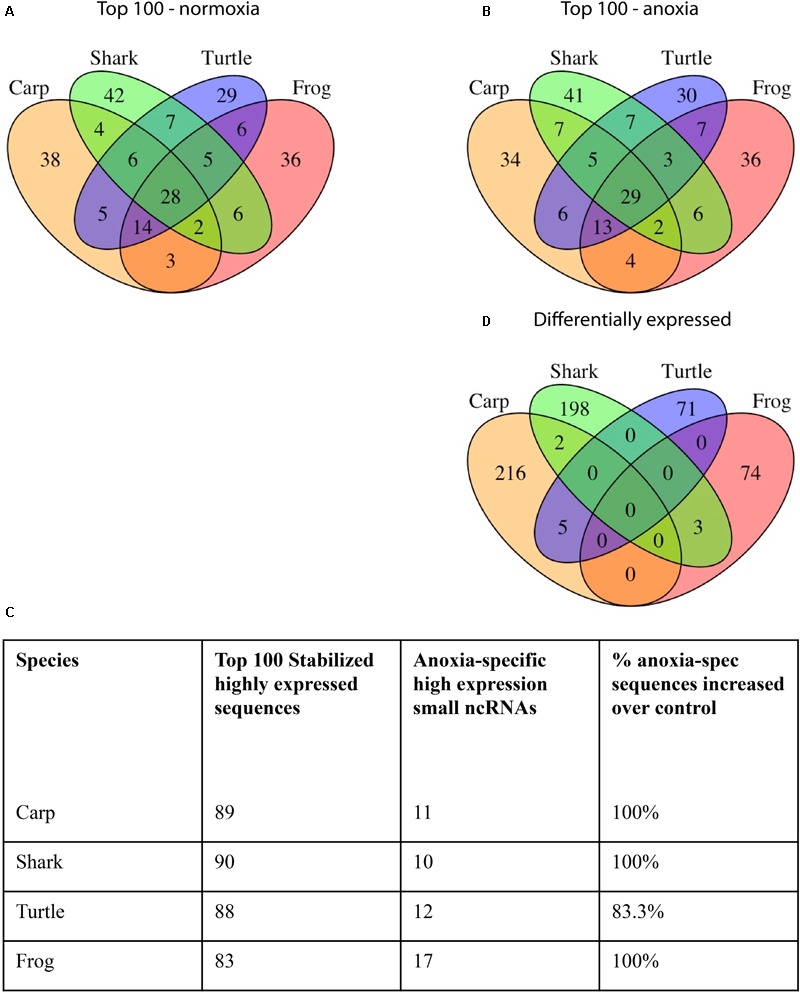
Venn diagram of small ncRNA sequences shared in brain tissue from each species. **(A)** The top 100 sequences most highly expressed at *t* = 0 (normoxia). **(B)** The top 100 sequences most highly expressed during anoxia. **(C)** Number of top 100 most highly expressed sequences under normoxia that are also in the list of top 100 most highly expressed sequences under anoxia. **(D)** Small ncRNAs that are differentially expressed in response to anoxia and recovery within each species. Sequences with 100% nucleotide matches were considered shared.

**FIGURE 4:**
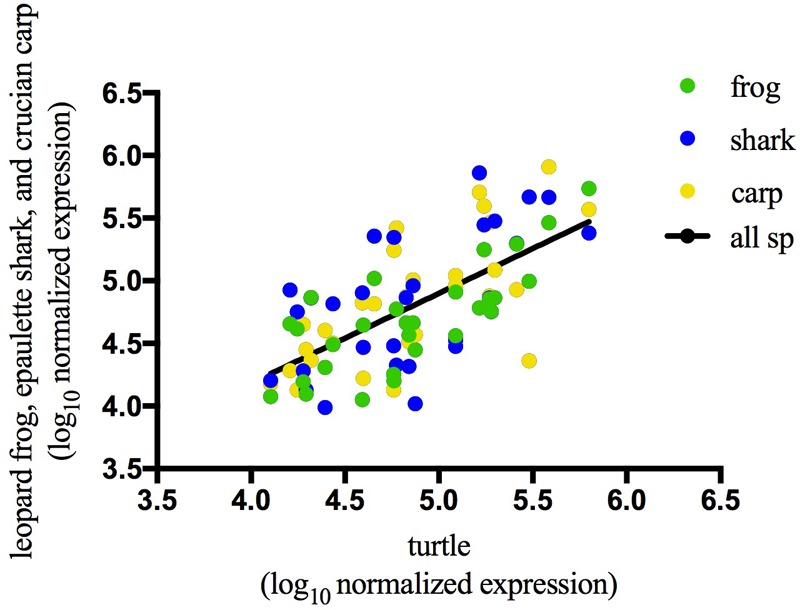
Correlation of normoxic expression values for the 29 sequences shared among the top 100 sequences from all 4 species. Log_10_ normalized expression values of top 100 most abundant small ncRNA sequences during normoxia are positively correlated with expression values of identical sequences in turtle during normoxia. *R*^2^ = 0.4494, *p* < 0.0001. See **Table 3** for correlations between all other pairs of species.

In the brains of the species studied, many abundant sequences were shared under normoxic conditions (**Figure 3A**) as well as during anoxia (**Figure 3B**). Most (80–90%) of the top 100 most highly expressed sequences under normoxia remained in the top 100 under anoxia (**Figure 3C**), indicating stabilization of these abundant sequences. This pattern also held true for the top 500 sequences (data not shown). Details on sequences highly expressed under anoxia but not under normoxia can be found in Supplementary Table S5. Of the top 100 most abundant sequences in each species under normoxia, 29 identical sequences were highly expressed in brain tissue of all four species (**Figure 3A** and **Table 2**), while additional sequences were found in common among only two or more species (**Figure 3A**). Of these exact sequence matches, 9 were also highly expressed under normoxia in embryos of the annual killifish, the most anoxia-tolerant vertebrate known (**Table 2**). Of the 29 shared highly expressed sequences, relative expression levels within each species was positively correlated with the expression levels of each other species (**Table 3** and **Figure 4**).

**Table 2:** Shared small ncRNAs identified in top 100 most abundant sequences under normoxia in all four species.

Small ncRNA sequence	Annotation simplified	Known response in literature	Top 100 most abundant in annual killifish embryos
TGAGGTAGTAGGTTGTATAGTTT	Let-7	Stress and hypoxia and preconditioning and stroke- responsive	
TGAGGTAGTAGGTTGTATAGTT^∗^			12 dpd embryos
TGAGGTAGTAGGTTGTATAGTT^∗^			

TTCAAGTAATCCAGGATAGGC^∗^	mir-26	Stress and hypoxia and stroke-responsive	
TTCAAGTAATCCAGGATAGGCT^∗^			

TGTAAACATCCCCGACTGGA^∗^	mir-30	Stress and hypoxia and preconditioning and stroke-responsive	
TGTAAACATCCCCGACTGGAAGCT			

TATTGCACTCGTCCCGGCCT^∗^	mir-92	Stress and hypoxia and preconditioning and stroke-responsive	
TATTGCACTCGTCCCGGCCTC^∗^			
TATTGCACTTGTCCCGGCCTGT^∗^			4 dpd and 12 dpd embryos

AACCCGTAGATCCGATCTTGTG^∗^	mir-99	Stress and preconditioning and stroke-responsive	

AACCCGTAGATCCGAACTTG^∗^	mir-100	Stress and stroke- responsive	
AACCCGTAGATCCGAACTTGT^∗^			
AACCCGTAGATCCGAACTTGTG^∗^			12 dpd embryos

TCCCTGAGACCCTAACTTGTG	mir-125	Stress and hypoxia and stroke-responsive	
TCCCTGAGACCCTAACTTGTGA^∗^			

CATTATTACTTTTGGTACGCG^∗^	mir-126	Hypoxia and stroke responsive	

TCACAGTGAACCGGTCTCTTT^∗^	mir-128	Stroke-responsive	4 dpd and 12 dpd embryos

TGAGATGAAGCACTGTAGCT^∗^	mir-143	Stress and stroke-responsive	

AACATTCAACGCTGTCGGTGA^∗^	mir-181	Stress and hypoxia and preconditionin g and stroke-responsive	4 dpd and 12 dpd embryos
AACATTCAACGCTGTCGGTGAG^∗^			
AACATTCAACGCTGTCGGTGAGT^∗^			
AACATTCATTGCTGTCGGTGGG^∗^			

TTCCCTTTGTCATCCTATGCCT^∗^	mir-204	Hypoxia and preconditioning and stroke responsive	4 dpd and 12 dpd embryos

AGCTACATCTGGCTACTGGGTCTC^∗^	mir-222	Stress and stroke-responsive	4 dpd and 12 dpd embryos

TCTTTGGTTATCTAGCTGTAT^∗^	Unknown		
TCTTTGGTTATCTAGCTGTATG^∗^			4 dpd and 12 dpd embryos
TCTTTGGTTATCTAGCTGTATGA^∗^			

TGAGGTAGTAGGTTGTATAGT	Unknown		

*Annotation information, stress response documented in literature, and presence in most abundant small ncRNAs in anoxia tolerant A. limnaeus embryos are included for each sequence (4 dpd, Wourms’ Stage 36 = metabolically active, high anoxia-tolerance; 12 dpd, Wourms’ Stage 40 = metabolically active, intermediate anoxia-tolerance). Asterisk indicates presence of small ncRNA in top 100 most abundant sequences under anoxia as well.*

**Table 3:** *R*-squared and *p*-values for linear regression of normalized expression values for the 29 sequences shared in high abundance (in top 100 most abundant small ncRNAs during normoxia).

	Western painted turtle	Crucian carp	Leopard frog	Epaulette shark
Western painted turtle		*r*^2^ = 0.4879	*r*^2^ = 0.5666	*r*^2^ = 0.3702
		*p* < 0.0001	*p* < 0.001	*p* < 0.0005
Crucian carp	*r*^2^ = 0.4879		*r*^2^ = 0.3064	*r*^2^ = 0.2909
	*p* < 0.0001		*p* = 0.0018	*p* = 0.0025
Leopard frog	*r*^2^ = 0.5666	*r*^2^ = 0.3064		*r*^2^ = 0.4099
	*p* < 0.0001	*p* = 0.0018		*p* = 0.0002
Epaulette shark	*r*^2^ = 0.3702	*r*^2^ = 0.2909	*r*^2^ = 0.4099	
	*p* < 0.0005	*p* = 0.0025	*p* = 0.0002	

*R^*2*^ values are positive and all slopes are significantly different from 0 for all comparisons*.

**FIGURE 5:**
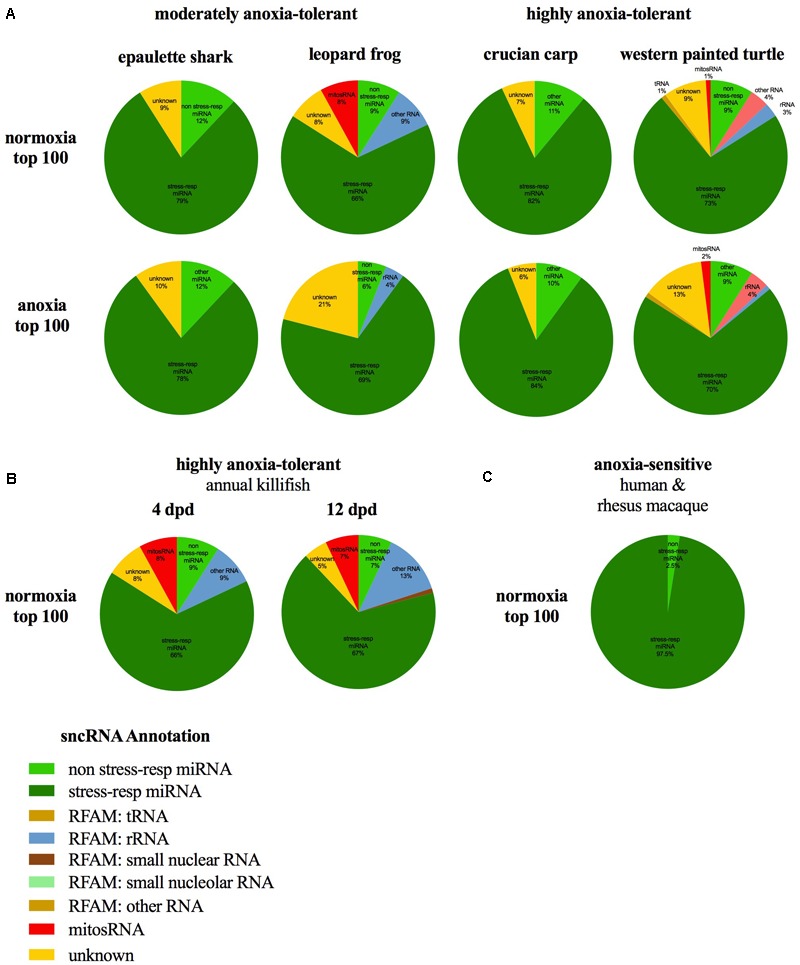
Annotation of abundant (top 100) small ncRNA sequences in normoxia, anoxia, and in other species of interest. Most small ncRNAs annotate to known stress-responsive miRNAs in the **(A)** epaulette shark, leopard frog, crucian carp, and painted turtle during normoxia and anoxia, **(B)** highly anoxia-tolerant annual killifish embryos, and **(C)** brain tissue of highly anoxia-sensitive species. Data for whole annual killifish embryos from Riggs and Podrabsky (2017). Data from Riggs and Podrabsky reanalyzed to present identical RNA subclass categories in this figure. Percentages for human and rhesus macaques based on top 40 miRNAs in brain (Shao et al., 2010).

Notably, many of the 100 most abundant sequences identified under normoxia and anoxia, including those shared among species, are known stress-responsive miRNAs in other species (**Figure 5A** and **Table 2**), meaning that their expression changes in response to stress. 25 of the 29 shared 100 most abundant small ncRNAs under normoxia are known stress-responsive miRNAs (**Table 2**). Of these 25 sequences, 22 are also highly expressed in all species under anoxia, again indicating stabilization of these sequences (**Table 2**). These miRNAs have been documented in the literature to increase or decrease in abundance in response to hypometabolism, hypoxia, ischemic-preconditioning, and stroke (Riggs and Podrabsky, 2017). This pattern is also shared in embryos of the annual killifish and anoxia-sensitive species (**Figure 5** and **Table 2**). Of the sequences shared between all four species and annual killifish embryos under normoxia, most sequences have been documented in the literature responding to more than one stressor (**Table 2**). Interestingly, however, many known stress-responsive miRNAs (exact sequence matches and variants) are also highly expressed in normoxic brains of anoxia-sensitive mammals such as humans and rhesus macaques (Shao et al., 2010; **Figure 5**). A meta-analysis of miRNAs highly expressed in these anoxia-sensitive species indicates that at least about half of the sequences that are highly expressed in the human brain are likely to change in response to anoxia, based on the changes in expression of these same sequence in hypoxia studies in hypoxia-sensitive cell lines (**Table 4**).

**Table 4:** Meta-analysis of abundant miRNAs and their predicted response in cell lines of anoxia-sensitive species.

miRNA	Abundance in human brain (Shao et al., 2010)	Predicted response to hypoxia (Kulshreshtha et al., 2008)
let-7f	18,832,757	Down
let-7g	7,578,064	Up and down
let-7a	5,117,974	Down
let-7c	4,516,955	Down
mir-103	1,303,040	Up
mir-101	857,034	Down
mir-107	768,588	Up
let-7i	584,441	Up
let-7e	562,093	Up and down
mir-7	371,940	Up
mir-181a	270,803	Up
let-7d	264,273	Down
mir-26a	251,367	Up
mir-125	221,344	Up
mir-191	143,704	Up
mir-26b	138,381	Up and down
mir-21	122,560	Up
mir-192	108,801	Up
mir-320a	91,968	Down

*Abundance values in human brain derived from Shao et al. (2010). All miRNAs listed were identified in the 40 most abundant small ncRNAs in the brain. The predicted response to anoxia for each miRNA listed is based on the response reported in hypoxia studies on cell lines of anoxia-sensitive species (Kulshreshtha et al., 2008). Note: only identical mature miRNA variants (denoted by the letter following the number in the miRNA name) were included in analysis*.

Although expression profiles of abundant small ncRNAs were broadly similar among species under normoxic conditions, and stabilized during anoxia, it should also be noted that between 30 and 41 of the top 100 most abundant sequences for each species were uniquely abundant in that single species under normoxia (**Figure 3A**). In each case, this number of unique small ncRNAs exceeded the number shared among all species studied, highlighting the diversity of the small ncRNA transcriptome across species, corroborating the species-specific clustering of samples by PCA.

### The Small ncRNA Response to Anoxia Varies by Species and Anoxia-Tolerance Level

#### Global Patterns of Differentially Expressed Sequences

**FIGURE 6:**
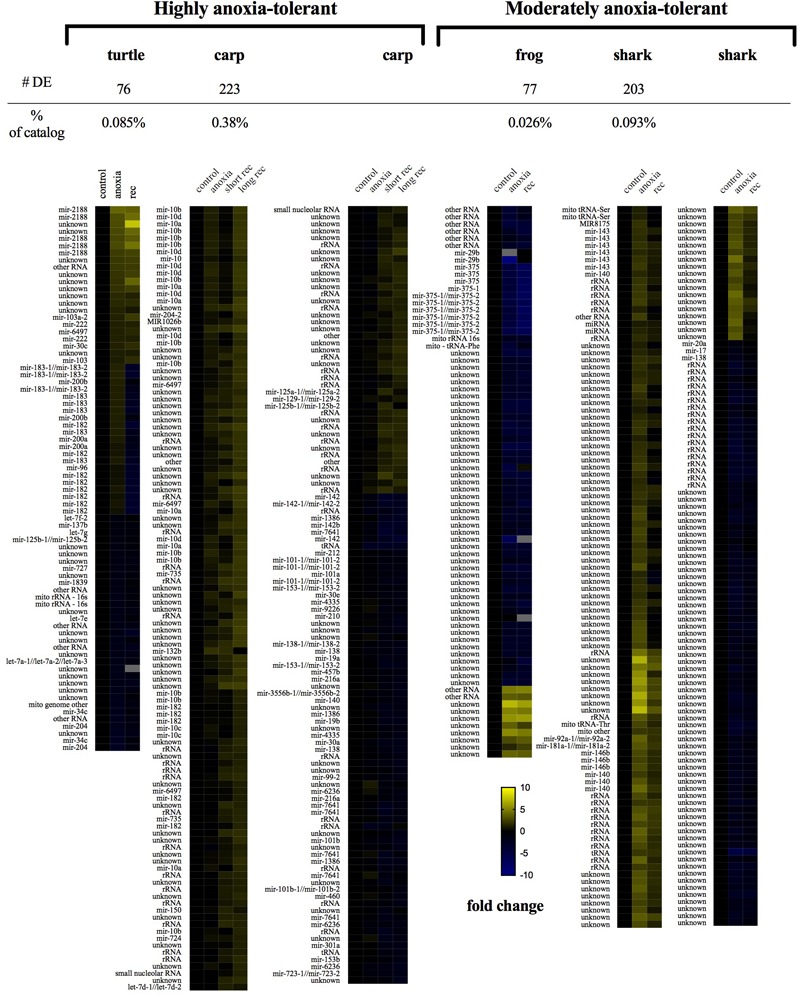
Heat maps of small ncRNA differential expression in response to anoxia and recovery from anoxia in each species studied. Highly differentially expressed sequences (adjusted *p*-value < 0.05, fold change > 2, and normalized mean expression across all samples > 25) were clustered within each species. Within each species, expression on the heat map corresponds with exposure to anoxia (middle column) and recovery (far right column). Log fold change expression values are set relative to *t* = 0 expression for each species (far left column). Yellow indicates an increase while blue a decrease in transcript abundance. Gray indicates a missing value.

**FIGURE 7:**
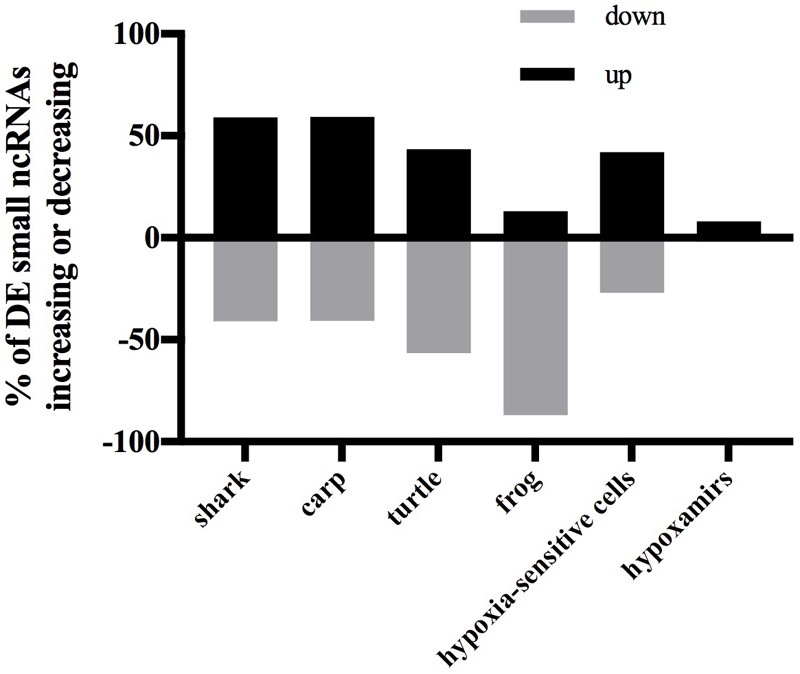
Bar chart showing percent of differentially expressed small ncRNAs that increase or decrease in response to anoxia. Black bars indicate an increase in expression from normoxia to anoxia and gray bars indicate a decrease in expression from normoxia to anoxia. Data for anoxia sensitive cells are from Kulshreshtha et al. (2008) and hypoxamirs are from Azzouzi et al. (2015).

**FIGURE 8:**
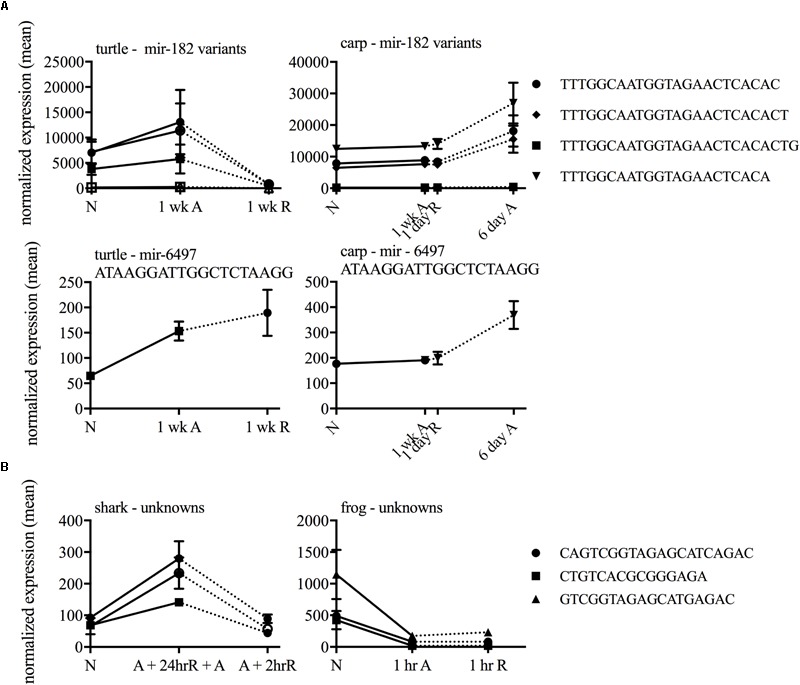
Line graphs of shared highly differentially expressed sequences among **(A)** highly anoxia tolerant species and **(B)** moderately anoxia tolerant species.

In each species only a small percentage (under 1%) of the total small ncRNAs identified in the species’ catalog changed statistically significantly in response to anoxia and recovery (**Figure 6**). In contrast to the normoxic small ncRNA profiles, anoxia and recovery elicited unique responses from the small ncRNA transcriptome in each species (**Figures 3D, 6**). The small ncRNA response of each species to anoxia varied in number of sequences differentially expressed, direction of change in expression, and identity of the sequences (**Figures 6, 7**). In each species, some sequences increased in abundance and some decreased in abundance. Notably, the majority decreased in abundance in the frog, while the majority in the studies of anoxia sensitive species increase in abundance in response to anoxia (**Figure 7**). No one sequence was differentially expressed among all anoxia-tolerant species studied (**Figure 3D**), and no more than 5 of the differentially expressed sequences for any given species were shared with another species (**Figure 3D**). Interestingly, five identical sequences were differentially expressed in the crucian carp and painted turtle, the most anoxia-tolerant representatives, but were not differentially expressed in the moderately anoxia-tolerant species, the shark and the frog (**Figure 3D**). Of these five sequences, four were variants of mir-182 (**Figure 8**). The other sequence annotated to mir-6497. In the crucian carp, these mir-182 sequences increased in abundance during late recovery, while in the turtle these same sequences increased in abundance during anoxia and dropped in abundance during recovery (**Figure 8A**). The shark and the frog share three identical sequences differentially expressed in response to anoxia and recovery (**Figure 3D**), but none of these annotated to known RNAs (**Figure 8B**). These sequences also differed in expression pattern, increasing in abundance during anoxia in the shark and decreasing in abundance during anoxia and recovery in the frog (**Figure 8B**).

#### Classes of Differentially Expressed Small ncRNAs

**FIGURE 9:**
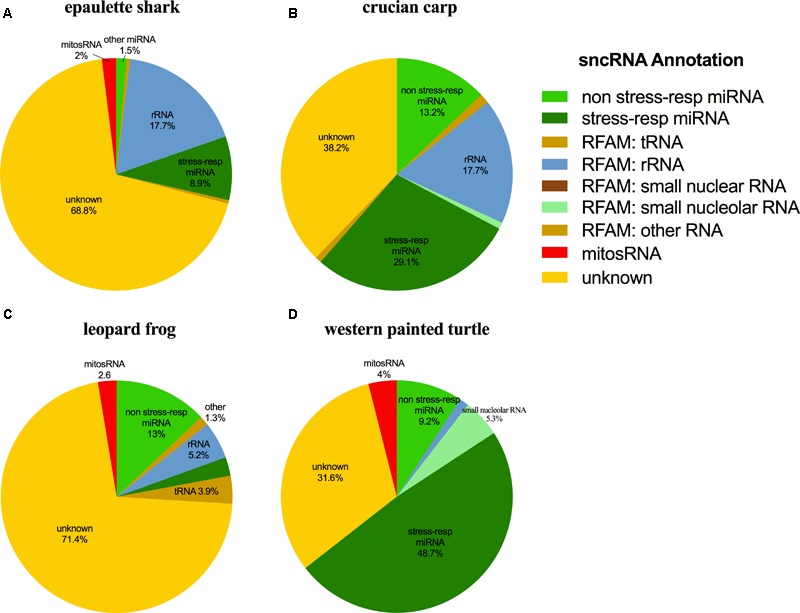
Annotation of differentially expressed small ncRNA sequences in each species. **(A)** Epaulette shark; **(B)** crucian carp; **(C)** leopard frog; **(D)** western painted turtle.

The identity of differentially expressed small ncRNAs varied among the species studied (**Figure 9**). In the crucian carp, 12.5% of the differentially expressed sequences annotated to variants of miR-10, while in the western painted turtle, 10.5% of the differentially expressed sequences annotated to miR-182, including sequences shared between the crucian carp and the turtle, and another 10.5% annotated to miR-183 (**Figure 6**). In the moderately anoxia-tolerant shark and frog, the majority of the differentially expressed sequences annotated to rRNA of other species or did not match any known sequences documented in miRBase or RFAM. In sum, each vertebrate studied had a distinct response at the small ncRNA level to anoxia and aerobic recovery in brain tissue.

**FIGURE 10:**
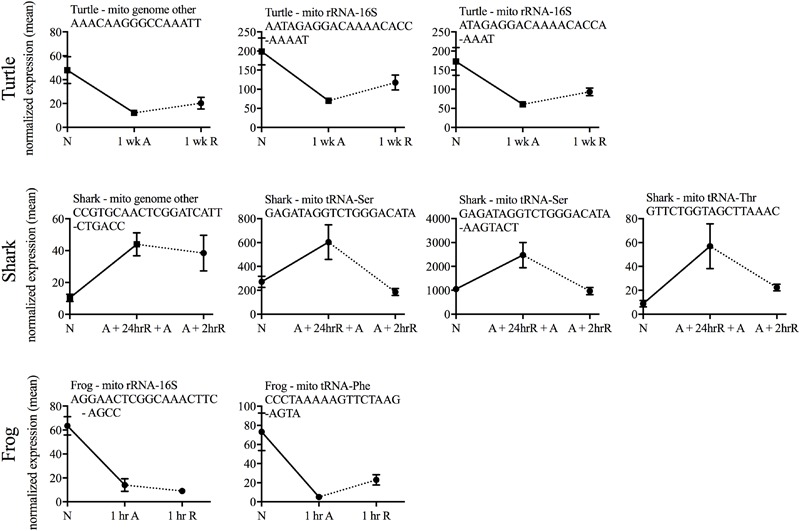
Line graphs of normalized mean expression of each differentially expressed putative mitosRNA identified within each species. No differentially expressed mitosRNAs were identified in carp. N, normoxia; A, anoxia; R, recovery.

**FIGURE 11:**
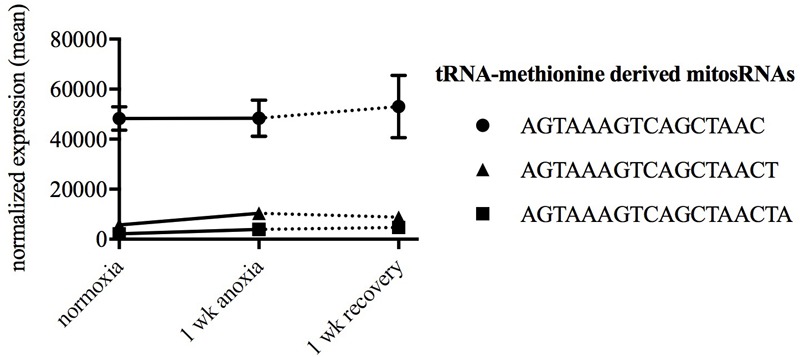
Line graph displaying normalized expression of the three most abundant mitosRNAs in turtles during normoxia. Each sequence is derived from mitochondrial tRNA-methionine. The data reveals a constitutive expression of these sequences in turtles during normoxia, anoxia, and recovery.

Putative mitochondria-derived small ncRNAs (mitosRNAs) were identified in the small ncRNA catalog of each species (**Figure 1**), and a few were highly differentially expressed in response to anoxia (**Figures 9, 10**). The number of mitosRNAs differentially expressed in each species was roughly proportional to the number of mitosRNAs identified in the catalog. In other words, there was no enrichment of mitosRNAs in the set of small ncRNAs differentially expressed in response to anoxia and recovery (compare **Figures 1, 9**). In the turtle, two sequences derived from mitochondrial 16S rRNA and one derived from an intergenic region of the mitochondria decreased in expression during anoxia, and increased in abundance during recovery (**Figure 10**). In the shark, four mitosRNAs were differentially expressed in response to anoxia, three of which were derived from tRNAs, all of which increased in abundance relative to control levels in response to anoxia (**Figure 10**). In the frog, two mitosRNAs decreased in abundance in response to anoxia: one derived from 16s rRNA and another derived from tRNA-Phe (**Figure 10**). Of note, several sequences derived from mitochondrial tRNA-methionine were abundant and constitutively expressed throughout exposure to anoxia and recovery in the turtle (**Figure 11**).

## Discussion

When faced with an extreme environmental challenge, such as anoxia, mechanisms for survival may include the organism’s physiological state under normoxia that predisposes them for tolerance, and an efficient and effective physiological response to oxygen deprivation. Species must make rapid changes to immediately adjust to anoxia, as well as changes to support long-term survival of anoxia. In this study, all four species respond to anoxia by a global stabilization of small ncRNAs expressed during normoxic conditions as illustrated by less than 1% of the sequences being differentially expressed. While many of the highly expressed sequences identified in anoxia tolerant species are shared with anoxia-sensitive species, it is the stabilization under anoxia that appears to distinguish the two groups and thus may represent a conserved mechanism for long-term survival of anoxia. Surprisingly, differential expression of small ncRNAs is unique in each species despite the high degree of sequence and functional conservation of many miRNAs across all vertebrates. These unique small ncRNA responses may reflect the fact that these species have evolved anoxia tolerance independently, and that adaptation to anoxia does not demand a common small ncRNA stress response. Below we discuss the patterns of small ncRNA expression observed in terms of the unique biological context for anoxia tolerance in the species investigated.

When evaluating the data presented here, it is important to consider some methodological differences among the species being compared. As a result of working with researchers and species from around the world, some different choices were made for each species concerning the exposure and sampling conditions. The turtle and shark were both acclimatized for winter, mimicking the winter conditions of their habitat at the time of exposure to anoxia, while the frog and carp were not. It is possible that these differences in exposure and sampling conditions could mask our ability to identify commonly differentially expressed genes. Another methodological difference is in the sampling exposure of the shark tissues. Sharks were exposed to anoxia until they lost their righting reflex, allowed to recover for 24 h, and then exposed to anoxia for 50 more minutes. While this sampling should reveal small ncRNAs induced by anoxia, it is possible that the second bout of anoxia results in expression changes of an additional set of sequences that was unaffected by the first exposure. Because these samples are extremely difficult to obtain, this was the only option for including a cartilaginous fish in this study. Additionally, the carp brains were stored in RNAlater^TM^ until RNA extraction, rather than being flash-frozen in liquid nitrogen, since the samples required international shipping. Although one cannot exclude that this sampling method contributed to some of the differences seen between the carp and the other anoxia-tolerant vertebrates, this appears unlikely since RNAlater^TM^ has been shown to adequately stabilize RNA for gene expression studies (Mutter et al., 2004), and is widely used for this purpose. Furthermore, the presence of identical sequences under normoxia in the carp and other species suggests that the carp samples can be compared to those of the other species, as it is unlikely that compromised samples would yield similar results. Finally, in two species whole brains were sampled (crucian carp and frog) while in the other two species only sub-regions of the brain were sampled (shark and turtle). This was again a matter of practicality in obtaining samples for the study. Since small ncRNAs can be tissue specific, it is possible that this would impair our ability to detect similar responses among species. However, many small ncRNAs are found in diverse tissues and a google scholar search of the top 10 most abundant miRNAs under normoxia for each species revealed literature documenting these miRNAs in an array of tissues. Additionally, PCA reveals clustering based on species, rather than experimental differences such as sampling temperature, brain region, or treatment, providing evidence that there was not a batch effect from any of these factors. Therefore, despite the methodological differences, we are confident that our methodologies can highlight robust responses to anoxia across all species, at least at the qualitative level. Thus, while there are limitations to what we can conclude from this work, we can still draw significant conclusions that inform us on the common and unique patterns of small ncRNA expression in anoxia tolerant vertebrates.

### Common and Stabilized Brain Small ncRNA Transcriptomes

A high proportion of stress-responsive miRNAs expressed under normoxia and stabilized during anoxia is a unifying feature of all four species. However, meta-analysis showed that stress-responsive miRNAs are also characteristic of brains of anoxia-sensitive species such as humans and rhesus macaques (Shao et al., 2010) where 39 and 38 of the top 40 sequences, respectively, annotated to known stress, hypoxia, or ischemia-responsive miRNAs. The fact that these miRNAs appear in brains of anoxia-tolerant and anoxia-sensitive species alike makes it difficult to determine if these sequences are playing a role in anoxia tolerance or preconditioning *per se*, or are simply a component of normal brain homeostasis. However, it is possible that these miRNAs may function differently in response to anoxia in the different species, such that in anoxia-sensitive species the expression pattern is one of dysregulation that does not support anoxia-tolerance, while in anoxia-tolerant species expression patterns support anoxia tolerance. The stabilization of these abundant small ncRNA sequences during anoxia may be a unifying principle that separates tolerant from sensitive species. However, detailed information on the expression of abundant small ncRNAs in the human and rhesus macaque in response to anoxia is lacking, and at this point we are restricted to using a meta-analysis to draw comparisons. Meta-analysis, however, does support the hypothesis that the highly expressed miRNAs in human brain are likely to change in abundance in response to anoxia. Ultimately, additional detailed studies in anoxia sensitive species may be required to fully evaluate this hypothesis.

Another possibility is that identical small ncRNAs may target different mRNAs in each species and still contribute to a common *physiological* response (e.g., metabolic depression) in different species. This hypothesis may be unlikely given the abundant evidence of conservation of miRNA sequence and function across diverse species (Ambros, 2004; Bartel, 2004). Therefore, further comparative studies with anoxia-sensitive species, identification of small ncRNA targets, and detailing their physiological function will be necessary to clarify the role of small ncRNAs in regulating a response to anoxia.

### The Small ncRNA Response to Anoxia and Recovery Differs Among Anoxia-Tolerant Vertebrates

Based on the findings of this study, there appears to be little data to support a conserved differential gene expression response for small ncRNAs that supports anoxia tolerance in vertebrate brain tissue. Quite the opposite, it appears that stabilization of normoxic levels of small ncRNAs may be of critical importance. The lack of a conserved induction of anoxia-specific or stress-specific small ncRNAs is somewhat surprising given the extreme nature of the stress, and the wealth of information on the conserved physiological mechanisms that support survival of anoxia, such as entry into a state of hypometabolism (Bickler and Buck, 2007). Further, a large existing body of literature on organismal and cellular responses to stress indicates that conserved cellular programs function in diverse organisms under a range of stressful conditions (Kultz, 2005). This deeply conserved stress response has been noted in a variety of anoxia-tolerant species at the levels of mRNA and protein expression (Storey, 2007; Kesaraju et al., 2009; Dowd et al., 2010; Stensløkken et al., 2010). Given these other well-described patterns of conserved stress responses, it is highly likely that a robust and conserved small ncRNA response to anoxia would have been identified in this study if it existed. Instead, these results suggest a highly nuanced response in differential expression at the small ncRNA level that may reflect species-specific responses to anoxia associated with differences in the level of anoxia tolerance, as well as the physiological, behavioral, and ecological context for surviving anoxia among the species studied. Interestingly, although there does not appears to be a master “anoxia tolerance miRNA,” the expression of some small ncRNAs was shared among organisms that displayed similar levels of anoxia tolerance: the highly anoxia tolerant carp and turtle, and the moderately anoxia tolerant shark and frog. Small ncRNAs identified from highly anoxia-tolerant species, such as the crucian carp and painted turtle, may have application for preventing or reversing the negative consequences of a heart attack or stroke.

### Small ncRNA Responses in Highly Anoxia-Tolerant Vertebrates

The most highly anoxia-tolerant adult vertebrates, the turtle and the crucian carp, display both common and distinct small ncRNA responses to anoxia in the brain. Both species depress their metabolism and reduce ATP turnover during anoxia, defending brain ATP levels and ionic gradients (Jackson, 1968; Nilsson, 2001). While anoxia and recovery induced changes in 5 identical small ncRNA sequences (4 annotating to miR-182 and one to miR-6497) in the turtle and the crucian carp, the patterns of expression are unique. Both of these sequences increase in abundance over anoxia and throughout recovery in the carp and increase during anoxia but decrease dramatically during recovery in the turtle. Interestingly, miR-182 is known to increase in abundance in response to ischemic preconditioning in the mouse brain (Lee et al., 2010). The pattern of miR-182 in the crucian carp, is consistent with a role during early recovery from anoxia, or anoxic preconditioning, while the pattern in the turtle supports a functional role during anoxia, but not during recovery. This is consistent with data suggesting that turtles may be “constitutively preconditioned” and therefore may lack a specific response to preconditioning (Lutz and Milton, 2004; Milton and Prentice, 2007; Warren and Jackson, 2017). In embryos of the annual killifish, identical miR-182 variants were expressed at high levels during normoxia in metabolically active, anoxia tolerant embryos, but anoxia did not increase miR-182 abundance in response to anoxia (Riggs and Podrabsky, 2017). The difference in expression pattern between the two highly anoxia-tolerant species suggests that they may have evolved related, yet distinct mechanisms for miR-182 and miR-6497’s involvement in anoxia tolerance.

One important difference between the crucian carp and other species studied is the muted magnitude of the small ncRNA transcriptomic response relative to the other species. Since the crucian carp does not enter as profound of a state of metabolic depression as the turtle (Jackson, 1968) and annual killifish embryos (Podrabsky and Hand, 2000; Fergusson-Kolmes and Podrabsky, 2007; Podrabsky et al., 2012) it is possible that less dramatic changes in gene expression maintain neural activity and support survival during anoxia. The crucian carp also has a robust small ncRNA response during recovery from anoxia compared to the turtle. Since the crucian carp is active when experiencing reoxygenation, it is possible that the threat of transient production of reactive oxygen species (ROS) and reperfusion injury is greater (Lefevre et al., 2017) compared to the turtle, which presumably recovers much more slowly from anoxia. Thus, we hypothesize that small ncRNAs may play a critical role in mediating survival during the transition out of anoxia in crucian carp, participating in the defense against reperfusion injury (Bickler and Buck, 2007; Stensløkken et al., 2014). Further evidence that small ncRNAs play an important role in preventing reperfusion injury comes from studies in embryos of the annual killifish (Riggs and Podrabsky, 2017). In the most anoxia tolerant and metabolically active stage of the annual killifish, the majority of small ncRNAs also increase in abundance during recovery from anoxia. Some of these sequences have been previously documented in the literature to play a role in regulating ROS production and toxicity. Interestingly, preconditioning does not extend the anoxia tolerance of these embryos, further indicating that these sequences may mediate the transition from anoxia back to normoxia, rather than in preconditioning the animal to better respond to subsequent anoxia.

### Small ncRNA Responses in Moderately Anoxia-Tolerant Species

Of the species studied, the frog and the shark are the least anoxia-tolerant. The frog brain slowly fails in the face of anoxia, showing a steady loss of ATP over 4–5 h that ultimately leads to neuronal depolarization (Milton et al., 2003). Compared to a mammal these death processes simply take more time in the frog (Knickerbocker and Lutz, 2001). Thus, the small ncRNA response of the frog may represent a gradual dysregulation or an inability to mount a small ncRNA response, as evidenced by the vast majority of the small ncRNAs decreasing in abundance during anoxia and recovery from anoxia. This contrasts mammals, where the majority of small ncRNAs responding to hypoxia increase in abundance during anoxia. Therefore this difference may be part of what separates the response to anoxia of frogs from mammals.

In contrast to the frog, the shark mounts what appears to be an active, regulated small ncRNA response, with nearly equal numbers of sequences increasing and decreasing in abundance in response to anoxia and recovery. Anoxia tolerance in the shark may be a byproduct of living in the cyclic hypoxic conditions of the Great Barrier Reef of Australia (Dowd et al., 2010). As such, the shark may be continuously preconditioned for survival of anoxia. Indeed, in contrast to the frog the epaulette shark maintains its brain ATP levels, at least for the first hour of anoxia (Renshaw et al., 2002). So, while the shark is also not highly tolerant, it may be in a very different physiological state from the frog and represent pathways of a more tolerant species. To illustrate this point, the frog and the shark differentially express three of the same sequences in response to anoxia and recovery, but all of them increase in abundance in the shark and decrease in the frog. Taken together, the anoxia-induced expression in the shark may support anoxia tolerance, while the pattern in the frog represents an inability to respond appropriately to anoxia.

In both the shark and the frog, the majority of differentially expressed small ncRNAs did not annotate to known RNAs. This is also true in the moderately anoxia-tolerant 20 dpd embryo of the annual killifish, where 35/65 differentially expressed small ncRNAs were unannotated (Riggs and Podrabsky, 2017). Given the relatively recent advent of small ncRNA sequencing, and its limited exploration in non-model organisms, novel sequences are not surprising. However, the higher proportion of unknown sequences in less anoxia-tolerant species may indicate novel evolutionary pathways for supporting intermediate anoxia tolerance, and may uncover novel therapies for preventing damage in the face of heart attack or stroke.

### Small ncRNA Subclasses and mitosRNA Expression

Within each species, miRNAs were the most abundant small ncRNA subclass and the majority of these miRNAs were known stress-responsive miRNAs. This pattern was also recently identified in embryos of the annual killifish (Riggs and Podrabsky, 2017). The proportion of differentially expressed sequences in each small ncRNA subclass varied among species. The domination of these sequences with miRNAs is notable, but many sequences remain uncharacterized, indicating a wealth of novel miRNAs or other small ncRNAs to explore in the context of anoxia tolerance.

An RNA subclass that has recently gained more attention, mitosRNAs, was of particular interest to examine and compare to the recent annual killifish small ncRNA study. In embryos of the annual killifish, mitosRNAs are highly differentially expressed in response to anoxia, and particularly increase in abundance during exposure to anoxia (Riggs and Podrabsky, 2017). While the small ncRNA catalog of each species here contained mitosRNAs, only a few sequences responded to anoxia or recovery in the turtle, shark, and frog, and none in the crucian carp. Very little is known about the biology of mitosRNAs. However, this study and the previous study on annual killifish embryos suggest that tRNA-derived fragments appear to be an important aspect of the mitochondrial small ncRNA transcriptome. The literature on nuclear-encoded tRNA-derived fragments consistently shows increased expression in response to stress (Saikia and Hatzoglou, 2015). This stress-responsive pattern appears to be conserved in mitochondrial tRNA fragments in embryos of the annual killifish (Riggs and Podrabsky, 2017) and brain of the shark. However, data presented here suggest a diversity of patterns for mitochondrial tRNA fragments. In the frog, mitochondrial tRNA-phenylalanine fragments decrease in response to anoxia, a pattern shared by mitochondrial tRNA fragments in turtles. In the frog, this may be a sign of dysregulation as discussed above, while in the turtle this decrease is more difficult to interpret. Turtles have a high constitutive level of a mitosRNA derived from tRNA-methionine compared to the other species. As speculated by Lutz and Nilsson (2004) and Warren and Jackson (2017) turtles may constitutively express molecular mechanisms that predispose them to tolerate anoxia. Perhaps mitosRNA-tRNA-methionine is one such component of these molecular mechanisms. Given the diversity of mitosRNA sequence expression patterns, determination of the species-specific roles that these small ncRNAs may play in the regulation of anoxia tolerance requires further experimentation.

In annual killifish embryos, there is compelling evidence that mitosRNAs play an important role in supporting anoxia tolerance. Anoxia and recovery induces changes in expression of many of these sequences, and the proportion of differentially expressed mitosRNAs is enriched compared to their representation in the total small ncRNA catalog (Riggs and Podrabsky, 2017). Therefore, the absence of a robust signal in the other anoxia-tolerant vertebrates studied here is interesting. Though there may be some similarity in the roles of mitosRNAs in anoxia tolerance among all the vertebrate species studied, they may also have tissue-specific or developmental roles in annual killifish embryos. Therefore, important next steps in understanding mitosRNA biology in anoxia tolerance include examining the expression of mitosRNAs in other tissues and developmental stages of the anoxia-tolerant species studied here. Additionally, for the leopard frog and the epaulette shark, mitochondrial genomes of closely related species were used to probe for putative mitosRNAs since mitochondrial genomes of those species were not available, which somewhat limits the analyses for those species. Further analysis of mitosRNAs in other anoxia-tolerant vertebrates will be necessary to better understand the scope of mitosRNAs in vertebrate anoxia tolerance. This area of research appears to be particularly promising given that recent literature suggests that mitochondrially derived small ncRNAs may influence mito-nuclear interactions (Pozzi et al., 2017). Thus, mitosRNAs may play an important role in communication flow between the oxygen sensing capabilities of the mitochondrion, and the coordination of a cellular response to oxygen deprivation.

## Conclusion

We have identified distinct small ncRNAs and expression patterns that may support anoxia tolerance in four major vertebrate lineages. Prior to anoxia exposure, anoxia-tolerant vertebrates display similar constitutive small ncRNA transcriptome profiles, and these profiles are stabilized in response to anoxia. Interestingly, many of these shared highly expressed sequences have already been documented in the literature to respond to stress/hypometabolism, hypoxia, ischemic preconditioning, or stroke in anoxia sensitive species. These data suggest a stabilization of small ncRNAs in anoxia tolerant species and a potential dysregulation in sensitive species. There does not appear to be a common set of anoxia-responsive small ncRNAs associated with vertebrate anoxia tolerance as evidenced by a lack of even a single sequence differentially expressed in all four anoxia-tolerant vertebrate species. However, common sequences were found within the highly anoxia tolerant species and within the moderately anoxia-tolerant species. These data are consistent with unique mechanisms evolving to support anoxia tolerance in each lineage. However, it is possible that some of these small ncRNAs target the same mRNA and feed into the same downstream pathways in the cell. Identification of the targets and functions of the anoxia-responsive small ncRNAs will be necessary to better understand the role of small ncRNAs in the evolution of anoxia-tolerance. Interestingly, however, the proportion of small ncRNAs differentially expressed in response to exposure to anoxia followed by recovery is very small (under 1%) for each species, suggesting a shared response where stabilization of abundant stress-responsive small ncRNAs is key. Additionally, when further examining the role of small ncRNAs in anoxia tolerance, it will be important to consider the timing of small ncRNA synthesis and degradation. Some small ncRNAs may be necessary for the immediate response to anoxia, while others may be central to supporting long-term survival of anoxia. The expression patterns of these sequences may vary as a result of these differences in biology. More detailed profiling of differential expression and biological function will be essential to developing a thorough understanding of the role of small ncRNAs in anoxia tolerance.

## Ethics Statement

The crucian carp study was carried out in accordance with the recommendations of the Norwegian Animal Research Authority. The protocol (#8400) was approved by the Norwegian Animal Research Authority. The leopard frog study was carried out in accordance with the recommendations of the Florida Atlantic University Institutional Animal Care and Use Committee. The protocol (#A15-11) was approved by the Florida Atlantic University Institutional Animal Care and Use Committee. The western painted turtle study was carried out in accordance with the recommendations of the Saint Louis University Institutional Animal Care and Use Committee. The protocol (#2198) was approved by the Florida Atlantic University Institutional Animal Care and Use Committee. The epaulette shark study was carried out in accordance with the recommendations of the Great Barrier Reef Marine Parks Authority. The permits (#G07/24973.1 and G07/23338.1) were approved by the Great Barrier Reef Marine Parks Authority.

## Author Contributions

CR and JP conceived of experiments. WD provided epaulette shark brain tissue samples from anoxia treatments. SM exposed frogs to anoxia and provided brain tissue. GN and SL exposed crucian carp to anoxia and provided brain samples. DW exposed western painted turtles to anoxia, provided brain tissue samples, and collected ancillary physiological data available in Supplementary Files. CR and AS extracted total RNA and prepared small RNA libraries for sequencing. CR conducted sequence data analysis. CR prepared the first draft of the manuscript. All authors contributed to and approved the final version of the manuscript.

## Conflict of Interest Statement

The authors declare that the research was conducted in the absence of any commercial or financial relationships that could be construed as a potential conflict of interest.
