# Supplementary material for: Small Non-coding RNA Expression and Vertebrate Anoxia Tolerance
Source: Front Genet. 2018 Jul 10;9:230. doi: 10.3389/fgene.2018.00230 (PMC6048248; doi:10.3389/fgene.2018.00230)
Supplement: Supplementary file 5 [file Table_5.DOCX]

Table S5. Sequence expression and annotation information for small ncRNAs present in top 100 most highly expressed sequences during anoxia but not in top 100 most abundant sequence during normoxia. Sequence information present for each species studied.

Western painted turtle

| **Sequence** | **control mean** | **anoxia mean** | **recovery mean** | **annotation** | **category** |
| --- | --- | --- | --- | --- | --- |
| AAGGTCCAACCTCACATGTCCT | 2,699.05 | 13,780.80 | 16,292.39 | mir-2188 | miRNA |
| TGACAACTCTTAGCGG | 4,792.60 | 13,169.29 | 11,817.27 | unknown | unknown |
| TTTGGCAATGGTAGAACTCACA | 7,039.14 | 13,077.61 | 791.94 | mir-182 | preconditioning |
| TTTGGCAATGGTAGAACTCACACT | 7,204.33 | 11,412.62 | 783.38 | mir-182 | preconditioning |
| TGAGGTAGTAGGTTGTATGGT | 9,038.90 | 10,967.32 | 12,803.49 | let-7c | stress & hypoxia & preconditioning |
| AGTAAAGTCAGCTAACT | 5,738.55 | 10,335.90 | 8,827.94 | tRNA-Met | mito-tRNA |
| TCTTTGGTTATCTAGCTGTA | 6,233.79 | 9,908.56 | 15,051.48 | unknown | unknown |
| ATACTGGGTGCTGTAGGCTT | 9,079.10 | 9,836.46 | 9,390.96 | unknown | unknown |
| ACCCCACTGCTAACTTTGACTGGTC | 7,076.72 | 9,631.47 | 7,627.64 |  | unknown |
| AAGCCCTTACCCCAAAAAGCAT | 8,125.78 | 9,575.37 | 12,915.67 | mir-129-2 | preconditioning |
| TAAGGCACGCGGTGAATGCC | 10,103.44 | 9,320.48 | 11,461.29 | unknown | unknown |
| TGACACAAGACTTTGT | 9,472.65 | 8,762.38 | 8,370.48 | unknown | unknown |

Crucian carp

| **sequence** | **control mean** | **anoxia mean** | **recovery 1 mean** | **recovery 2 mean** | **annotation** | **category** |
| --- | --- | --- | --- | --- | --- | --- |
| ACCCTGTAGAACCGAATTTGT | 11,870.58 | 25,248.27 | 13,574.58 | 48,941.16 | mir-10b | stress |
| TAAAGCTAGAGAACCGAATGTAT | 11,627.14 | 20,302.47 | 13,597.86 | 23,341.21 | mir-9-3 | stress |
| TAGCAGCATGTAAATATTGGAGT | 8,798.71 | 18,178.79 | 12,139.68 | 23,683.54 | mir-16c | stress & hypoxia |
| TAGCAGCATGTAAATATTGGAG | 12,176.94 | 17,916.48 | 19,255.01 | 20,935.96 | mir-16c | stress & hypoxia |
| TGAGAACTGAATTCCATAGATGG | 7,781.13 | 17,789.41 | 6,884.34 | 17,461.69 | mir-146a | hypoxia & preconditioning |
| TCGTACCGTGAGTAATAATGCA | 12,197.66 | 17,090.21 | 15,223.96 | 18,744.44 | mir-126a | hypoxia |
| CATTGCACTTGTCTCGGTCTGA | 11,006.24 | 17,080.50 | 15,611.09 | 18,241.22 | mir-25 | stress |
| TGAGGTAGTAGTTTGTATAGT | 12,740.30 | 14,309.34 | 16,580.49 | 16,865.61 | let-7g | stress & hypoxia & preconditioning |
| TATTGCACTTGTCCCGGCCTGTA | 12,659.61 | 14,090.79 | 17,880.80 | 14,109.39 | mir-92a-1 | stress & hypoxia & preconditioning |
| TATTGCACTCGTCCCGGC | 10,499.03 | 13,966.04 | 11,686.82 | 16,054.93 | mir-92 | stress & hypoxia & preconditioning |
| TTTGGCAATGGTAGAACTCACA | 12,472.17 | 13,331.65 | 14,027.92 | 26,968.83 | mir-182 | preconditioning |

Epaulette shark

| **Sequence** | **control mean** | **anoxia mean** | **recovery mean** | **annotation** | **category** |
| --- | --- | --- | --- | --- | --- |
| TGAGATGAAGCACTGTAGCTC | 7,126.63 | 24,357.36 | 15,461.48 | mir-143 | stress |
| TGAGATGAAGCACTGTAGCTCT | 5,610.63 | 21,740.40 | 13,318.88 | mir-143 | stress |
| CAGTCGGTAGAGCATC | 2,620.25 | 19,633.69 | 2,696.61 |  | unknown |
| CAGTGCAATATTAAAAGGGC | 6,966.30 | 12,634.40 | 9,212.14 | mir-130c | stress |
| TATTGCACTTGTCCCGGCCTGTAT | 7,750.17 | 12,531.06 | 9,037.74 | mir-92a-1 | stress & hypoxia & preconditioning |
| TGCTCAGTAGTCAGTGTAGATTC | 7,887.93 | 12,469.50 | 9,886.27 | mir-222a | stress |
| TGAGGTAGTAGGTTGTATAGTA | 7,419.37 | 11,648.32 | 7,701.64 | let-7 | stress & hypoxia & preconditioning |
| ATCCCGGACGAGCCCCCA | 8,361.61 | 11,517.33 | 8,669.45 | mir-5103 | miRNA |
| TTCAAGTAATCCAGGATAG | 7,194.50 | 11,325.40 | 10,391.24 | mir-26-1//mir-26-2//mir-26-3 | stress & hypoxia |
| TAAGGCACGCGGTGAATGCC | 6,693.34 | 10,202.04 | 8,400.44 |  | unknown |

Leopard frog

| **Sequence** | **control mean** | **anoxia mean** | **recovery mean** | **annotation** | **category** |
| --- | --- | --- | --- | --- | --- |
| GCCCAAAATCTAGCCTTCAAGACT | 14.65 | 25,405.93 | 12,017.59 |  | unknown |
| GTAAAACCTGCCGGGACT | 9,093.64 | 22,409.05 | 15,304.40 |  | unknown |
| TGAGAACTGAATTCCATGGACTGT | 9,617.94 | 17,058.89 | 14,300.27 | mir-146b | hypoxia & preconditioning |
| TTTGGCAATGGTAGAACTCACACT | 10,127.37 | 16,061.83 | 19,360.39 | mir-182 | preconditioning |
| ACCGTGGCTTTAGATTGTTACT | 9,662.55 | 14,548.42 | 11,887.53 | mir-132a | stress & hypoxia |
| TCAGACCTCAGATCAGACGCGGCGACC | 7,213.71 | 14,307.01 | 9,516.58 | X59734.1/3092-7391 | rRNA |
| AACATTCATTGCTGTCGGTGGGT | 9,728.58 | 14,176.83 | 11,426.39 | mir-181b-1//mir-181b-2 | stress & hypoxia & preconditioning |
| AACATTCGACGCTGTCGGTGAG | 8,473.46 | 13,427.30 | 7,537.46 | mir-181a-1//mir-181a-2 | stress & hypoxia & preconditioning |
| AACATTCAACGCTGTCGGTG | 6,366.64 | 13,355.50 | 8,564.57 | mir-181a-1//mir-181a-2 | stress & hypoxia & preconditioning |
| GGGAATACCAGGTGCTGTAGGCTT | 4,201.84 | 12,905.46 | 18,690.89 |  | unknown |
| TCAGACCTCAGATCAGACGCGGC | 6,011.80 | 12,485.16 | 8,931.37 | X59734.1/3092-7391 | rRNA |
| CGTTGTAGGCCTCTCCAGCACT | 8,876.72 | 12,385.02 | 11,796.11 |  | unknown |
| TATTGCACTCGTCCCGGCCTCCA | 9,428.24 | 12,285.02 | 8,795.11 | mir-92b | stress & hypoxia & preconditioning |
| AACATTCAACGCTGTCGGTGG | 8,193.06 | 12,217.10 | 11,148.33 | mir-181a | stress & hypoxia & preconditioning |
| TGAGGTAGTAGATTGTATAGTTA | 8,679.68 | 11,904.39 | 8,416.53 | let-7f-1//let-7f-2 | stress & hypoxia & preconditioning |
| TTTGGCAATGGTAGAACTCACA | 8,507.92 | 11,887.68 | 35,445.05 | mir-182 | preconditioning |
| AACATTCATTGCTGTCGGTGA | 5,920.87 | 11,841.25 | 9,736.92 | mir-181b | stress & hypoxia & preconditioning |
