# Supplementary material for: Small Non-coding RNA Expression and Vertebrate Anoxia Tolerance
Source: Front Genet. 2018 Jul 10;9:230. doi: 10.3389/fgene.2018.00230 (PMC6048248; doi:10.3389/fgene.2018.00230)
Supplement: Supplementary file 6 [file Data_Sheet_1.DOCX]

**Turtle methods for analysis of blood**

Immediately after the brain dissection, and with the turtle’s neck still clamped, the subclavian artery was catheterized with intramedic PE90 tubing for sampling of arterial blood into a heparinized 1cc syringe. Arterial blood gases and pH were determined by injecting 0.3 mL of the sample into a BMS3 Mk2 system thermostatted to 3°C, and measurements were read from a Radiometer PHM73 pH/blood gas analyzer (Radiometer, Copenhagen, Denmark). Blood pH was determined using an Orion 4 Star pH meter paired with a flexible Accumet Microprobe^TM^electrode (Fisher Scientific, Pittsburgh, PA) placed at the bottom of the center well where injected blood flows after it exits the gas measurement cell. This method provides pH measurements directly comparable to those acquired in previous studies of painted turtles(13). Plasma bicarbonate was calculated using the Henderson-Hasselbalch equation with α = 0.0812 (37), and pK’ = 6.293, when pH > 7.97, or pK’ = 6.350, when pH < 7.97 (14, 36).

For plasma metabolite analysis, blood was centrifuged at 10,000 *g* for 3 min at 3°C in a Sorvall Legend Micro 17 Centrifuge (Thermo Scientific, Waltham, MA). Plasma was immediately aliquoted into tubes and flash-frozen in a slurry of ethanol and dry ice for storage at -80°C for later analysis. Plasma lactate and glucose were measured using a YSI 2300 STAT Plus (YSI Incorporated, Yellow Springs, OH, USA). Hematocrit was determined by collecting blood in heparinized capillary tubes and centrifuging for 3 min at 13,000 *g* in an IEC MB Micro Hematocrit Centrifuge (Thermo Scientific, Bellport, NY). Data available in Table S1.
