# Supplementary material for: Small Non-coding RNA Expression and Vertebrate Anoxia Tolerance
Source: Front Genet. 2018 Jul 10;9:230. doi: 10.3389/fgene.2018.00230 (PMC6048248; doi:10.3389/fgene.2018.00230)
Supplement: Supplementary file 7 [file Image_1.pdf]

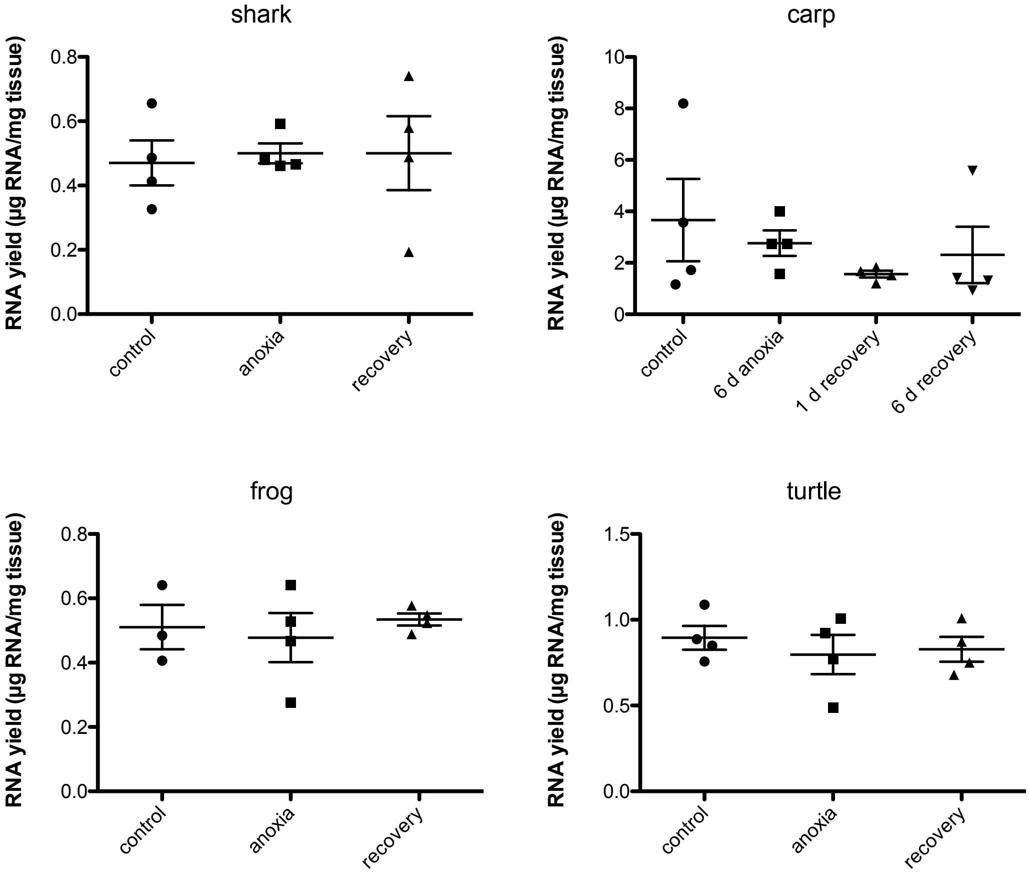

Figure S1. RNA yield per gram of brain tissue for each animal during each treatment. Total RNA does not differ in response to anoxia and recovery within each species: shark (ANOVA, p=0.9538; Tukey's post hoc p > 0.05), carp (ANOVA, p=0.5328; Tukey's post hoc p > 0.05), frog (ANOVA, p=0.7851; Tukey's post hoc p > 0.05), turtle (ANOVA, p=0.7320; Tukey's post hoc p > 0.05).
