# Supplementary figures and images for: Small Non-coding RNA Expression and Vertebrate Anoxia Tolerance
Source: Front Genet. 2018 Jul 10;9:230. doi: 10.3389/fgene.2018.00230 (PMC6048248; doi:10.3389/fgene.2018.00230)

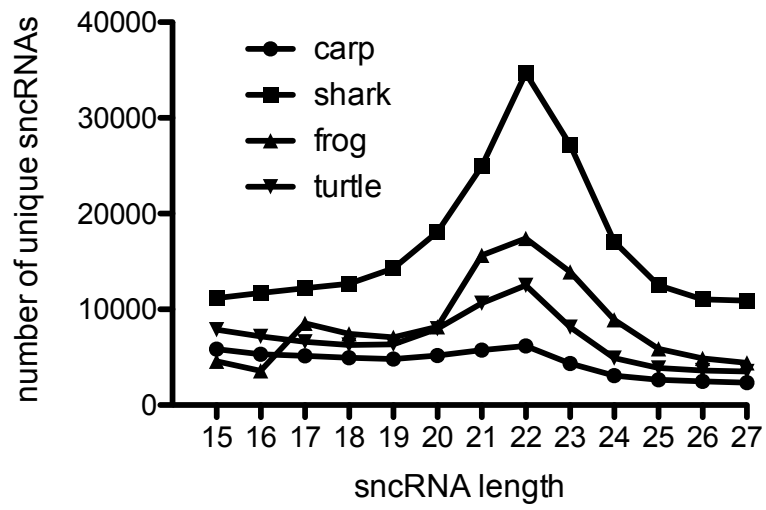

**Figure S2.** Length distribution of unique small RNAs identified in each species.

Supplement: Supplementary file 8 [file Image_2.pdf]
